# The association of *CD36* variants with polypoidal choroidal vasculopathy compared to typical neovascular age-related macular degeneration

**Published:** 2012-01-18

**Authors:** Hiroaki Bessho, Shigeru Honda, Naoshi Kondo, Sentaro Kusuhara, Yasutomo Tsukahara, Akira Negi

**Affiliations:** Department of Surgery, Division of Ophthalmology, Kobe University Graduate School of Medicine, Chuo-ku, Kobe, Japan

## Abstract

**Purpose:**

To clarify the association of cluster of differentiation 36 (*CD36*) variants with polypoidal choroidal vasculopathy (PCV) and compare them with those in typical neovascular age-related macular degeneration (tAMD).

**Methods:**

We included 349 Japanese AMD patients (210 PCV, 139 tAMD) and 198 age-matched controls. Four tag single-nucleotide polymorphisms (SNPs)—rs10499862, rs3173798, rs3211883, and rs3173800—in the *CD36* region were genotyped using the TaqMan assay. Allelic and genotypic frequencies of the SNPs were tested.

**Results:**

Although none of the SNPs tested were associated with PCV, the allelic frequencies of rs3173798 and rs3173800 were significantly different between PCV and tAMD patients. Genotype association analysis demonstrated different associations of these two SNPs between PCV and tAMD in the genotype model. Haplotype analysis revealed that the association of the major haplotype (T-T-T-T) at four selected SNPs in CD36 differed significantly between PCV and tAMD patients.

**Conclusions:**

The *CD36* region may be associated with the difference in genetic susceptibility for PCV and tAMD.

## Introduction

Age-related macular degeneration (AMD) is a leading cause of central vision loss in the elderly in industrialized countries [1]. Although the number of patients with AMD has increased remarkably over the years, the pathology of AMD is not well understood [2]. Advanced AMD is clinically classified into atrophic AMD and exudative AMD. In exudative AMD, there are two characteristic phenotypes distinct from typical neovascular AMD (tAMD), which are called polypoidal choroidal vasculopathy (PCV) [3,4] and retinal angiomatous proliferation [5]. These two phenotypes are known to have different aspects from tAMD in their natural courses, as well as different responses to interventions such as photodynamic therapy (PDT) and anti–vascular endothelial growth factor therapy, although the reasons for this remain unknown [6-10]. Recently, several genetic association studies have been conducted to explain the different characteristics among the phenotypes of exudative AMD and their susceptibility to several interventions, mainly PDT [11-19].

Cluster of differentiation 36 (CD36) is a multifunctional molecule that plays an important role in lipid metabolism, angiogenesis, inflammation, and scavenging oxidative stresses [20-22], all of which may be involved in the pathogenesis of AMD and in the mechanism whereby PDT functions. We previously demonstrated the association of coding variants in the *CD36* region with the incidence of neovascular AMD (corresponding to tAMD in the present report) in the Japanese population [23]. In that study, two variants of single nucleotide polymorphisms (SNPs)—rs3173798 and rs3211883 on introns 3 and 4, which have high linkage disequilibrium—showed the greatest association with susceptibility to neovascular AMD. However, it has not been determined whether this association is general to all exudative AMD or specific for tAMD. We hypothesized that the genetic variants in *CD36* may be differently associated with genetic susceptibilities to tAMD and PCV. Since PCV is known to have a better response to PDT than tAMD [7,8], we considered that the scavenging ability of CD36 for reactive oxygen species generated by PDT might be different between tAMD and PCV, which could influence the effect of PDT.

We previously reported that coding variants of the elastin gene (*ELN*) were associated with PCV but not with tAMD [13]. However, two recent reports with larger cohorts showed the opposite results: The *ELN* polymorphism was associated with tAMD but not with PCV [24,25]. Although the association of elastin gene variants with tAMD and PCV is still inconclusive, these results might have been generated due to statistical type 1 and type 2 errors. In the present study, we included a sufficient number of subjects based on power analysis to prevent these errors.

In this study, we genotyped four tag SNPs located on introns 3 and 4 of the *CD36* gene, and analyzed the association between these variants and the incidence of PCV, as well as tAMD, in a Japanese population.

## Methods

### Study participants

This is an extension study of a previous report [23]. The study was approved by the Institutional Review Board of the Kobe University Graduate School of Medicine, and was conducted in accordance with the Declaration of Helsinki. Written informed consent was obtained from all subjects. All cases in this study were Japanese individuals recruited from the Department of Ophthalmology at Kobe University Hospital in Japan.

The study included 349 Japanese AMD patients (210 PCV, 139 tAMD) and 198 age-matched controls who accepted DNA sampling. The tAMD group in this study included the patients of our previous study, along with 30 new patients. The control group in this study included the subjects of the previous study and 16 more individuals [23]. All patients received ophthalmic examinations, including visual acuity measurements with refractive correction, slit-lamp biomicroscopy of the fundi, color fundus photographs, optical coherence tomography, fluorescein angiography, and indocyanine green angiography (ICGA). All PCV subjects enrolled in this study met the criteria of definite cases of PCV as proposed by the Japanese Study Group of Polypoidal Choroidal Vasculopathy [26]. ICGA showed a choroidal origin of the polypoidal lesions in all PCV cases, typically with vascular networks in the posterior poles and subretinal reddish-orange protrusions corresponding to the polypoidal lesions on ICGA. In contrast, all tAMD patients had clear images of choroidal neovascular networks on ICGA. The classification of the AMD phenotype was performed by three independent retinal specialists for each case under masked conditions for the genotype. Only those cases whose diagnoses were matched by all three readers were included in this study. The details of the participants are listed in Table 1.

**Table 1:** Data summary of PCV and tAMD patients and control subjects.

**Factors**	**PCV**	**tAMD**	**Control**
Number of subjects	210	139	198
Gender (male/female)	166/44	108/31	119/79
Mean age±SD (years)	73.8±7.5	75.3±7.3	72.1±5.9
Median age (years)	75	76	72
Age range (years)	51–93	55–94	56–95

PCV: polypoidal choroidal vasculopathy, tAMD: typical neovascular AMD.

### Single-nucleotide polymorphism selection

The four tag SNPs—rs10499862, rs3173798, rs3211883, and rs3173800 on introns 3 and 4 in the *CD36* region—were selected based on our previous study [23], including SNPs which were significantly associated with tAMD (allelic nominal p-values <0.01). These four SNPs were in a single haplotype block using the algorithm based on the solid spine of linkage disequilibrium.

### Genotyping

Genomic DNA was extracted from the peripheral blood using the QIAamp DNA Blood Maxi Kit (Qiagen, Valencia, CA). Genotyping was performed using TaqMan^®^ SNP Genotyping Assays or Custom TaqMan^®^ SNP Genotyping Assays (Applied Biosystems, Foster City, CA) on a StepOnePlus™ Real-Time PCR System (Applied Biosystems) in accordance with the supplier’s recommendations.

### Statistical analysis

All SNPs were evaluated for Hardy–Weinberg equilibrium using the χ^2^ test (one degree of freedom) with SNPAlyze version 7.0.1 (DYNACOM, Yokohama, Japan). The allelic and genotype frequency distributions were compared among tAMD, PCV, and control subjects using a χ^2^ test with one or two degrees of freedom for the allelic and genotypic tests, respectively. To avoid false-positive results, the Bonferroni or permutation correction was added for each comparison. P values <0.05 were considered to be statistically significant.

To exclude a potential stratification, our study cohort was examined by Structure 2.0 [27] using 26 unlinked genome-wide SNPs, as shown in our previous report [23].

## Results

None of the SNPs reported in the present study showed any significant deviations from the Hardy–Weinberg equilibrium over the entire sample (p>0.05). Table 2 summarizes the minor allelic frequencies for all SNPs and the results from a single-SNP association study. Significant differences in minor allelic frequencies were found in all SNPs tested between the tAMD and control subjects, as shown in our previous report [23], but there was no difference in minor allelic frequencies between the PCV and control subjects. Meanwhile, significant differences were found between PCV and tAMD patients at rs3173798 and rs3173800. The statistical powers of single-SNP association analysis at rs3173798 and rs3173800 were about 0.94 and 0.92, respectively (alpha error <0.05), in the comparison between PCV and tAMD. The genotype association analysis revealed significant associations of rs3173798 and rs3211883 with tAMD, but not with PCV in the dominant model (Table 3). In addition, the most significant differences were found between PCV and tAMD patients at rs3173798 and rs3173800 in the genotype (additive) model. In the haplotype analysis, the most frequent haplotype (T-T-T-T) at rs10499862, rs3173798, rs3211883, and rs3173800 showed the most significant difference in association between PCV and tAMD (Table 4). There was no evidence of significant stratification in our study cohort (*Pr* [*K*=1 >0.99]).

**Table 2:** Summary of single-SNP association analysis on gene *CD36*.

			**Minor allele frequency**			**Allelic nominal p-value (Bonferroni p-value)**		
**SNP ID**	**Location**	**Major/ Minor**	**PCV**	**tAMD**	**Control**	**PCV versus control**	**tAMD versus control**	**PCV versus tAMD**
rs10499862	Intron 3	T/C	0.14	0.11	0.18	0.058 (0.23)	0.0066 (0.026)	0.28 (1.0)
rs3173798	Intron 3	T/C	0.46	0.33	0.44	0.57 (1.0)	0.005 (0.020)	0.00081 (0.0032)
rs3211883	Intron 4	T/A	0.35	0.28	0.4	0.19 (0.76)	0.0017 (0.0068)	0.043 (0.17)
rs3173800	Intron 4	A/T	0.26	0.38	0.28	0.41 (1.0)	0.0071 (0.028)	0.0005 (0.0020)

SNP: single nucleotide polymorphism, PCV: polypoidal choroidal vasculopathy, tAMD: typical neovascular AMD.

**Table 3:** Summary of the genotype association analysis.

		**Genotype frequency (%)**			**Genotype association results**					
		**Genotype (Major homo/Hetero/Minor homo)**			**PCV versus Control (dominant model)**		**tAMD versus Control (dominant model)**		**PCV versus tAMD (genotype model)**	
**SNP ID**	**Major/ Minor**	**PCV**	**tAMD**	**Control**	**OR (95%CI)**	**Nominal p value**	**OR (95%CI)**	**Nominal p value**	**Nominal p value**	Corrected p value*
rs10499862	T/C	75/22/3	81/17/2	67/29/4	0.58 (0.19–1.80)	0.34	0.52 (0.14–2.01)	0.34	0.5	0.52
rs3173798	T/C	28/51/21	45/44/11	36/40/24	0.8 (0.50–1.28)	0.36	0.41 (0.22–0.75)	0.0034	0.0031	0.0033
rs3211883	T/A	42/45/13	52/40/8	40/41/19	0.59 (0.35–1.02)	0.059	0.36 (0.18–0.74)	0.0038	0.13	0.14
rs3173800	A/T	52/44/4	37/50/13	54/36/10	0.35 (0.15–0.82)	0.012	1.32 (0.67–2.61)	0.42	0.00067	0.00069

Corrections were performed with permutation test. SNP: single nucleotide polymorphism, PCV: polypoidal choroidal vasculopathy, tAMD: typical neovascular AMD, OR: odds ratio, CI: coefficient interval.

**Table 4:** Haplotype-based association study.

		**Frequency**				**Permutation P**		
**Index**	**Haplotype**	**Overall**	**PCV**	**tAMD**	**Control**	**PCV versus control**	**tAMD versus control**	**PCV versus tAMD**
H1	T-T-T-T	0.32	0.26	0.38	0.28	0.45	0.01	0.001
H2	T-T-T-A	0.28	0.28	0.28	0.27	0.82	0.8	0.97
H3	T-C-A-A	0.19	0.21	0.17	0.21	0.85	0.27	0.2
H4	C-C-A-A	0.15	0.14	0.11	0.19	0.06	0.005	0.25
H5	T-C-T-A	0.05	0.11	0.06	0.05	0.001	0.63	0.018

Selected SNPs are rs10499862/rs3173798/rs3211883/rs3173800. SNP: single nucleotide polymorphism, PCV: polypoidal choroidal vasculopathy, tAMD: typical neovascular AMD.

## Discussion

We genotyped four tag SNPs in the *CD36* region, and found that the minor allelic frequencies at SNPs rs3173798 and rs3173800 and the haplotype at four selected SNPs in the *CD36* region were significantly associated with the difference in genetic susceptibility to PCV and tAMD. Namely, T, T alleles were less frequent than C, A alleles in PCV than in tAMD patients at rs3173798 and rs3173800, respectively. Moreover, the T-T-T-T haplotype at rs10499862, rs3173798, rs3211883, and rs3173800 on introns 3 and 4 in the *CD36* region was significantly less frequent in PCV than in tAMD.

Although our previous report demonstrated an association of SNPs in the *CD36* region with neovascular AMD [23], the phenotype specificity of these SNPs remained to be elucidated. The present study suggests a different association of *CD36* in genetic susceptibility for PCV and tAMD, which may contribute to the different clinical characteristics of PCV (i.e., natural course and the response to PDT or ranibizumab) from tAMD [3,4,6-9]. Since the SNPs at rs10499862, rs3173798, rs3211883, and rs3173800 were not covered by the gene chips used in previous genome-wide association studies [28,29], they could not be detected as possible causative SNPs for AMD in those studies. The present study, as well as our previous report [23], further suggested an association of this region in *CD36* with the incidence of tAMD. However, these SNPs did not remain significant in the prevalence of PCV. Moreover, a statistically significant difference was detected in the association of this region between tAMD and PCV. This suggests a different association of the *CD36* region with the phenotype of neovascular AMD, although the details have not yet been clarified. A recent in vivo study demonstrated that a downregulation of *CD36* in capillary sprout endothelial cells facilitated angiogenesis [30]. Rats carrying a specific genetic variant of *CD36* have been found to be more susceptible to light-induced retinal damage, and are more likely to develop age-related retinal degeneration and choriocapillary rarefaction [31]. CD36 is involved in diverse physiologic and pathological processes, including scavenger receptor functions, transforming growth factor-β activation, lipid metabolism, angiogenesis, atherogenesis, and inflammation, depending on the ligands with which CD36 can interact [20-22]. In particular, the scavenging ability of CD36 for oxidative stress is critical to manage AMD, since oxidative stress is widely recognized as an important component in the pathogenesis of AMD [32,33] and in the mechanism whereby PDT works to occlude neovascular tracts [34]. A recent in vitro study reported that the uptake of oxidized low-density lipoprotein (oxLDL) induces the expression of several genes related to oxidative stress, inflammation, and apoptosis in retinal pigment epithelium cells [35]. An immunohistochemical study reported the presence of oxLDL in surgically excised choroidal neovascularization membranes [36]. Moreover, the verteporfin used in PDT binds with serum LDL, and this complex is incorporated into choroidal neovascularization tissues [37]. Although it is not known whether lipid metabolism and oxidative stress play different roles in the pathogenesis between tAMD and PCV, the present study implied different genetic susceptibilities for each AMD phenotype. Interestingly, recent reports demonstrated a difference between tAMD and PCV in their histological findings and systemic risk factors [38,39]. Moreover, our previous study demonstrated a different association of an elastin gene polymorphism with tAMD and PCV [13].

The biologic basis of the association with the haplotype in *CD36* is currently unknown, because the haplotype in the present study does not reside in the coding sequence of *CD36*. FASTSNP [40] gave the information that rs3173798 is located at splicing site with medium-high effect, but it was not shown whether it is located at splice donor site or acceptor site [41]. Thus, the SNPs in this region could have noncoding effects on gene expression and function. A recent study demonstrated that the C allele at rs3173798 tended to increase *CD36* expression while reducing high-density lipoprotein levels [41,42]. Since the C allele at rs3173798 was less frequent in the tAMD group than the PCV and control groups, the reduced expression of *CD36* might be correlated with tAMD but not PCV pathogenesis. Moreover, Picard et al. have recently demonstrated an accumulation of oxLDL in Bruch’s membrane among aged *CD36* knockout mice [43]. The oxLDL accelerates an accumulation of deposits in Bruch’s membrane and causes drusen. Interestingly, drusens are more frequently seen in tAMD than PCV [44]. However, exhaustive resequencing of this locus may elucidate potentially undiscovered and more important causative variants. In addition, it is essential to perform replication studies using other cohorts to verify and conclude the associations of *CD36* variants with tAMD and PCV.

The limitation of this study was a possible influence of lipid metabolism on AMD pathogenesis [45,46]. Since CD36 is known to associate with the metabolic syndrome and hyperlipidemia [41,47], *CD36* variants may indirectly contribute to AMD via abnormal lipid metabolism. However, the results of this study suggest a possible role of lipid metabolism in the different pathogeneses between PCV and tAMD.

In conclusion, the present study suggested some clinical possibilities for genetic association analysis that can be further investigated to determine the specific pathogenesis of PCV as distinct from that of tAMD.
